# The function of copulatory plugs in *Caenorhabditis remanei*: hints for female benefits

**DOI:** 10.1186/1742-9994-7-28

**Published:** 2010-11-02

**Authors:** Nadine Timmermeyer, Tobias Gerlach, Christian Guempel, Johanna Knoche, Jens F Pfann, Daniel Schliessmann, Nico K Michiels

**Affiliations:** 1 Department of Animal Evolutionary Ecology, Institute for Evolution and Ecology, University of Tuebingen, Auf der Morgenstelle 28, 72076 Tuebingen, Germany

## Abstract

**Background:**

Mating plugs that males place onto the female genital tract are generally assumed to prevent remating with other males. Mating plugs are usually explained as a consequence of male-male competition in multiply mating species. Here, we investigated whether mating plugs also have collateral effects on female fitness. These effects are negative when plugging reduces female mating rate below an optimum. However, plugging may also be positive when plugging prevents excessive forced mating and keeps mating rate closer to a females' optimum. Here, we studied these consequences in the gonochoristic nematode *Caenorhabditis remanei*. We employed a new CO_2_-sedation technique to interrupt matings before or after the production of a plug. We then measured mating rate, attractiveness and offspring number.

**Results:**

The presence of a mating plug did not affect mating rate or attractiveness to roving males. Instead, females with mating plugs produced more offspring than females without copulatory plugs.

**Conclusions:**

Our experiment suggests that plugging might have evolved under male-male competition but represents a poor protection against competing males in our experiment. Even if plugging does not reduce mating rate, our results indicate that females may benefit from being plugged in a different sense than remating prevention.

## Background

The limitation of male fitness by access to oocytes drives competition between males [1,2] and selects for a variety of traits that aid males in accomplishing fertilization. Preventing female partners from remating by means of mating plugs is one option to reduce competition with rival males [3-5]. Mating plugs represent a physical barrier between a female's oocytes and the sperm of possible future male partners. They vary in composition from mucous, gelatine like substances in bumble bees and nematodes [3,6] to more solid, coagulated protein mixtures in primates [7,8] or even whole appendages as in spiders where a males' pedipalp can break off and remain lodged in the female genital opening [9]. Mating plugs have also been shown to reduce female attractiveness [10-12] and receptivity [10-12] and can work as sperm reservoirs [13]. These documented advantages of mating plugs for males are assumed to outweigh the costs of producing a plug or losing a pedipalp to seal the female's gonopore. It is therefore not surprising that mating plugs have been documented for a broad range of animal phyla, including insects [11,14], arachnids [9], reptiles [10,12,13,15], and rodents [16].

Whatever the function of the plug from the male's perspective is, it will always directly or indirectly influence female fitness. Reduced mating rates could, for example, lead to female sperm depletion and thereby reduce fertility. Alternatively, mating plugs may reduce exposure to harassment or physical harm caused by subsequent males. In this case, a plug would not only represent a conflict between the sexes, but should also be seen as a sexually mutualistic effect induced by the male. Finally, the plug itself could be a nutrition gift from the male to increase female -and therefore also male- fitness. In insects, these gifts are widely spread as reviewed by Gwynne [17] and can be nuptial, oral and seminal and in that sense also be provided by a copulatory plug. For *Drosophila hibisci *Polak *et al. *[5] tested if females digest copulatory plugs presumably to increase fitness but did not find any effect of their treatments.

In terrestrial nematodes, copulatory plugs consist of gelatinous mass, whose genetic background is investigated in Palopoli *et al*. [6]. On the behavioural level, plugging has to date been investigated in different strains of *Caenorhabditis elegans*. Barker [18] showed that *C. elegans *produces a gelatinous plug. This plug mainly increased the chances of losing contact to a female [18], but it did not prevent intromission of the male's spicules. The plug in *C. elegans *also does not affect sperm precedence of previous males [19]. Hence, available evidence from *C. elegans *remains inconclusive about the putative role of plugging in male-male competition; other functions are also possible.

In addition to its function as a (weak) physical barrier [18] the plug could reduce female attractiveness. Mature *Caenorhabditis *females produce a male-attracting pheromone, which is not species-specific but mating history specific [20,21]. Females lose their attractiveness immediately when mating but regain it within several hours post mating [20], which coincides with the onset of egg laying and the associated loss of plugging substance. This suggests that pheromone release could be negatively affected by a mating plug, although females also release pheromones through the skin [21]. Finally, a plug may contain chemicals that stimulate egg production, reduce female receptivity or attractiveness to other males, or enhance the uptake and storage of the plugging male's sperm.

In *C. elegans*, hermaphrodites that copulate with males suffer from reduced lifespan [22] and reduced fitness [23] suggesting a cost of mating resulting from a conflict between the sexes. Also for *Caenorhabditis remanei *there is evidence that female fecundity is reduced due to increased mating rates [24]. Therefore, it is conceivable that mating plugs in *Caenorhabditis *nematodes might lower this mating rate and therefore influence female fecundity and fertility positively.

**Figure 1:**
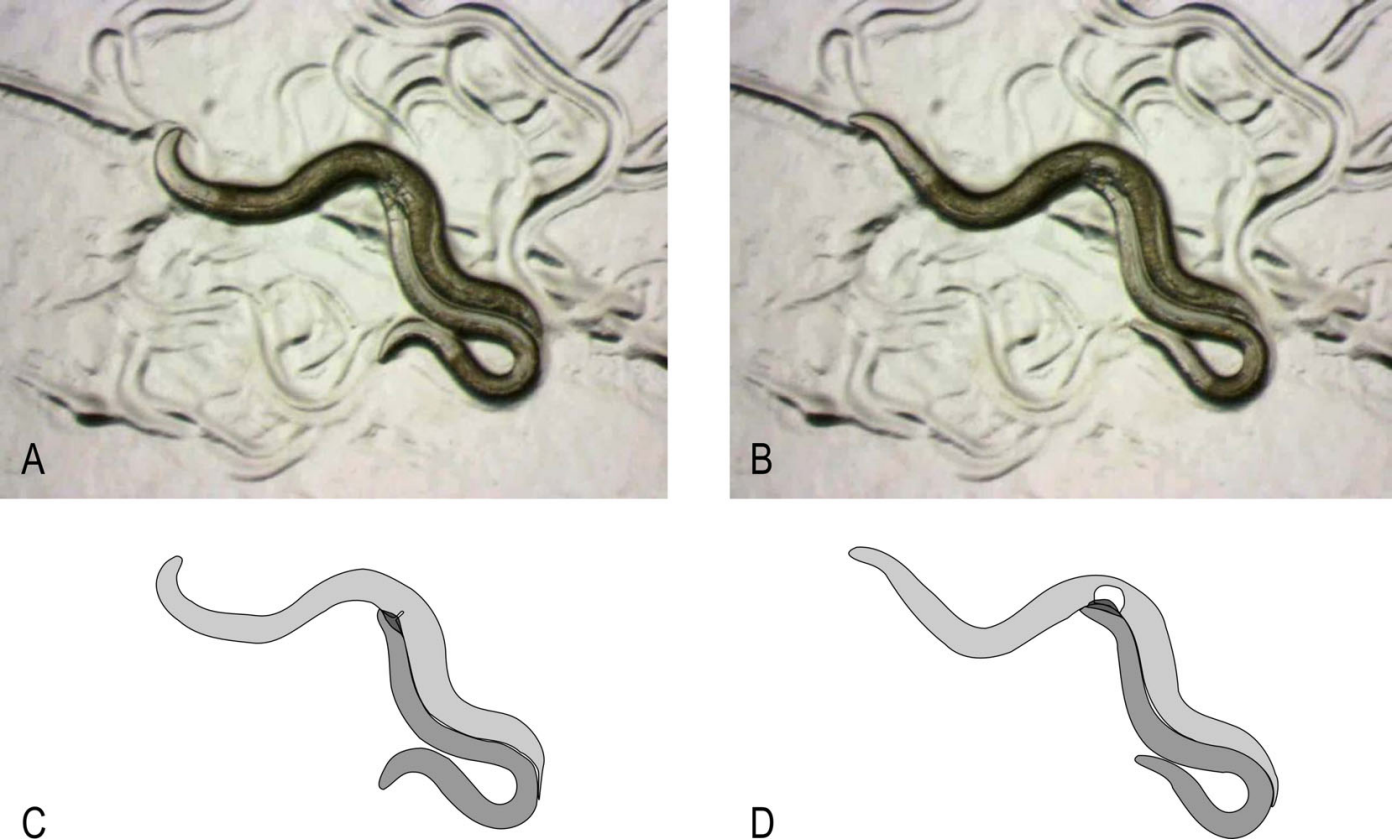
**Mating in *C. remanei. ***A pair of *C. remanei *before (A) and after sperm transfer (B). The male (medium grey, C) covers the female vulva with its fan (dark grey). After ejaculation, a cloud of sperm (white, D) is visible in the female (light grey) genital tract.

**Figure 2:**
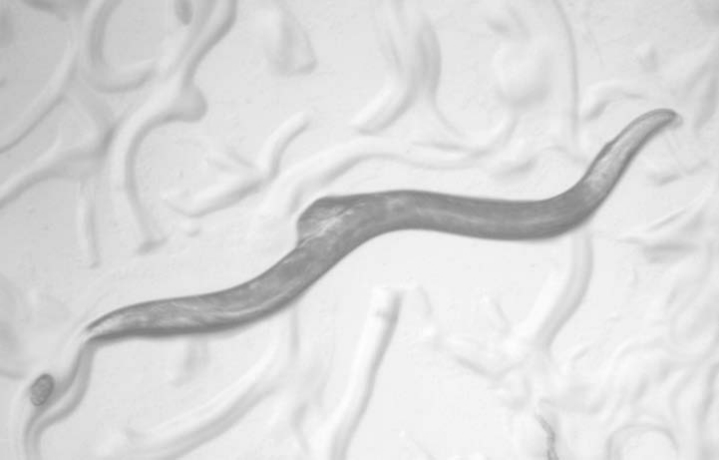
**Plugged female of *C. remanei***. Males of *C. remanei *produce a gelatinous substance after mating, which is visible as a translucent cap over the female's vulva.

Here, we tested the effects of male mating plugs in the gonochoric nematode *C. remanei*. The species has a life-cycle of 3 days at 20°C. Mate searching is very efficient in *C. remanei *compared to *C elegans *[25]; a single male and virgin female placed together in a 3 cm Petri dish typically find each other within 10 min (personal observation). The male then usually starts scanning the female's body to locate the vulva. It inserts its spicules, a paired, sickle-shaped structure positioned at the male's genital opening. At this point the female stops moving and insemination starts immediately (Figure 1). Copulation ends with plugging; the male transfers a transparent substance produced in a gland near the genital opening onto the female's vulva, which polymerizes directly [own observation, see also: 26, 27]. Figure 2 shows a plugged female of *C. remanei*. Females mate on average 23.4 times during their reproductive phase from day 3 to day 6 in a 5:5 mating group on a 3 cm Petri dish as we tested in a pilot study. Sperm of different males are assumed to accumulate between the uterus and the oviduct as in *C. elegans *[28], leading to post-copulatory sperm competition. Maturing oocytes are fertilized at this site before being transferred to the uterus and laid through the vulva.

Palopoli *et al*. [6] tested whether the existence of a plug prevents a female from direct remating. In their study, they found a difference between plugged and unplugged females; the presence of a plug prevented direct remating and therefore can lead to reproductive advantage of the first male.

The purpose of this study is to assess whether plugging is not just in the male's interest, but also increases female fitness by keeping mating frequency below a level at which male coercive mating becomes harmful. We also expected that a copulatory plug lowers mating frequency and attractiveness to males, possibly as a consequence of reduced pheromone release.

We tested these hypotheses by comparing two treatment groups. In the "unplugged" group, males were removed during copulation, shortly after sperm transfer, but before plugging, whereas in the "plugged" group, males were removed after a complete copulation sequence, including plugging. Using this procedure, focal females were mated 18 times with new, non-virgin males of the same age within three consecutive days. Effects of a plug on female detection and attractiveness were assessed by measuring the time until the first contact, the time from contact to mating and the overall mating rate. Contact and mating events as well as the number of eggs and offspring were compared between the two treatments.

## Results

**Figure 3:**
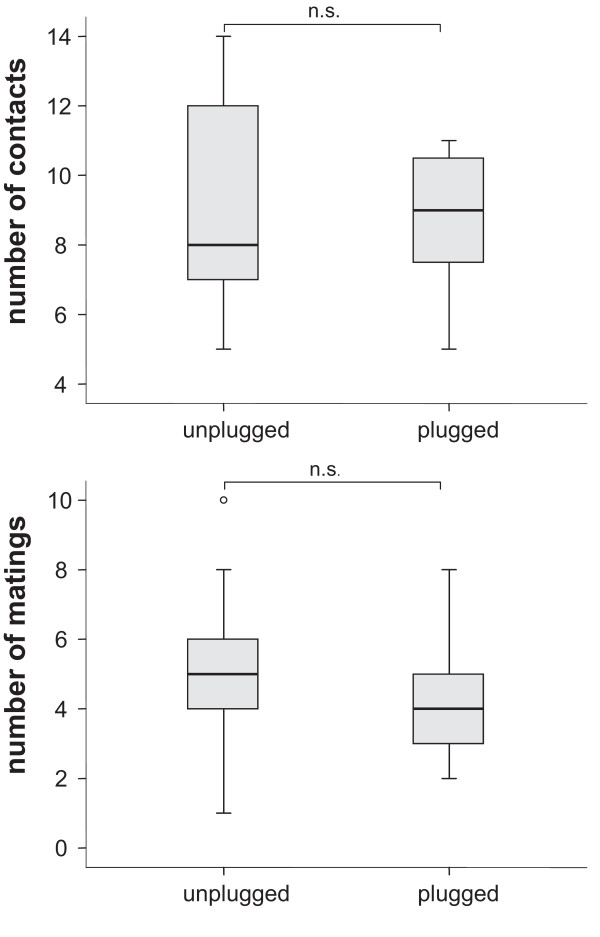
**Box-plot of the number of contacts and subsequent matings in unplugged and plugged females**. Data per replicate female (n = 24 in each group) are the sum across 18 consecutive mating opportunities. There was no significant difference, neither in contact frequencies (Wilcoxon rank sums test: *Z *= -0.09; *p *= 0.93), nor in mating frequencies (Wilcoxon rank sums test: *Z *= -1.18; *p *= 0.24).

**Figure 4:**
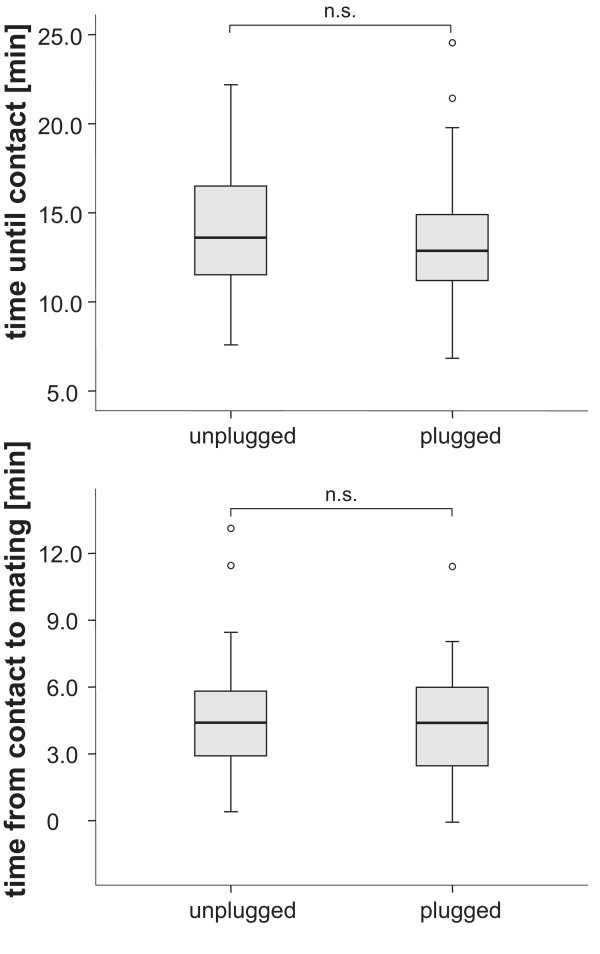
**Box-plot of the average delay between the start of each mating trial to first contact, and from first contact to actual mating in unplugged and plugged females**. The difference was not significant, neither for the delay until first contact (Wilcoxon rank sums test: *n*_*plugggd *_= 24; *n*_*unplugged *_= 24; *Z *= -0.57; *p *= 0.57), not for the time between first contact and mating (Wilcoxon rank sums test: *n*_*plugged *_= 24; *n*_*unplugged *_= 22; *Z *= 0.51; *p *= 0.61).

The number of body contacts as well as the total number of matings did not differ significantly between plugged and unplugged females (Figure 3). In both treatments, the females had 18 mating opportunities for 30 minutes each. Females in the plugged treatment had on average 8.7 ± 1.8 contacts and 4.3 ± 1.6 matings whereas females of the unplugged treatment contacted on average 9.0 ± 2.8 times and had 5.0 ± 2.0 matings. In addition, neither the time until first contact nor the time from first contact to mating differed between treatments (Figure 4).

**Figure 5:**
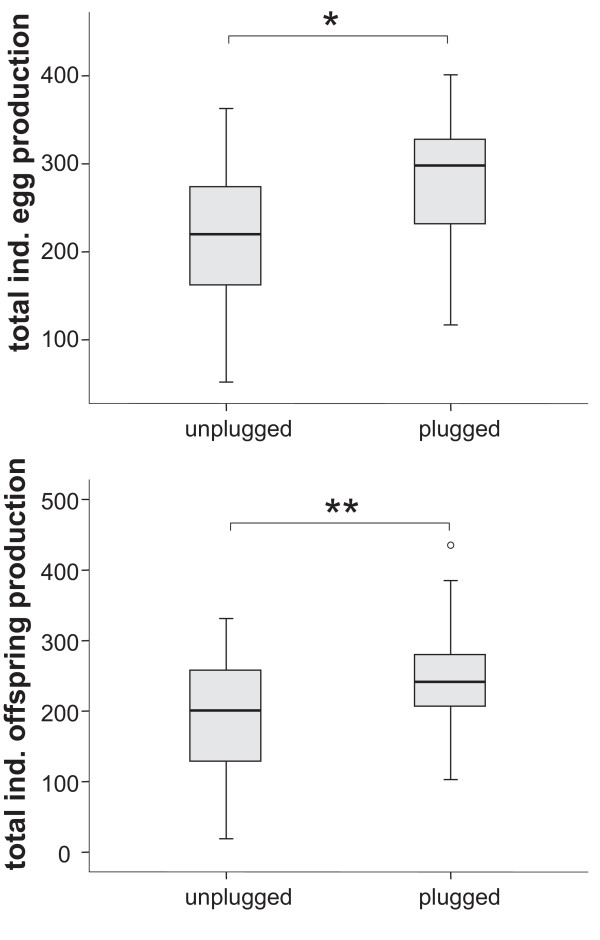
**Box-plots showing fecundity (total egg production) and fitness (total adult offspring) in unplugged and plugged females**. Plugged females produced significantly more eggs (*Wilcoxon rank sums test*: *n*_*plugged *_= 22; *n*_unplugged _= 23; *Z *= -2.66; *p *= 0.008) as well as offspring (*Wilcoxon rank sums test*: *n*_*plugged *_= 24; *n*_*u*__nplugged _= 24; *Z *= 2.25; *p *= 0.025) than unplugged females.

In contrast, plugged females laid on average 29% more eggs in the three experimental days than unplugged females (282 ± 69 versus 218 ± 81 eggs, respectively, Figure 5a). As a consequence, also the number of hatchlings of plugged females exceeded that of unplugged females by about 29% (252 ± 85 versus 195 ± 92 offspring, Figure 5b).

Hatching rate, which we define as fertility (see Methods), did not differ between treatments (*Wilcoxon rank sums *test: *n*_*plugged *_= 22; *n*_*unplugged *_= 23; *Z *= 0.238; *P *= 0.8).

## Discussion

Our results indicate that, in *C. remanei*, plugging does neither affect the likelihood that a female is located by males, nor whether or not mating ensues. However, we found that plugging has a significant positive effect on egg production, suggesting that plugs may represent a beneficial act of a male towards its female partner rather than a competitive act between males. Note that this does not exclude the possibility that plugs may have acted repulsively against other males when arising in the course of evolution.

We can see three reasons for the here apparent lack of an effect on female remating rate and therefore in male-male competition compared to other studies dealing with plugging in *Caenorhabditis *[6,18]. First, the plug could offer services which are in the interest of both the male and female. For instance, a plug may act as a seal, keeping sperm inside the female. In our treatments we did not see sperm leakage after matings that were interrupted before plugging, but it has been shown in *C. elegans *[29]. The plug may also contain substances that stimulate the female. From *Drosophila *it is known that males trigger the female to invest more resources in the offspring of this particular male [30]. Hodgkin and Doniach [19] showed that in *C. elegans *plugging does not affect the relative fitness of plugging versus non-plugging males, so these presumed substances could have general effects that are not exclusively beneficial to the male producing them. Other substances, such as nutrition or antimicrobial additives, can also lead to higher male fitness in a way that is not in conflict with the interests of the female. Female partners might also benefit from a physical barrier preventing pathogens from entering the body via the vulva. For example, *Drechmeria coniospora*, a fungus that parasitizes nematodes, can invade a female worm through the vulva [31]. A mating plug may seal the vulva and protect gametes and embryos.

Second, females were paired to one male at a time, preventing an immediate, second mating attempt by a second male. Our study design does not exclude such immediate effects on female receptivity in the first hour after plugging in contrast to the behavioural study provided in the supplement to Palopoli *et al. *[6]. Here the females were confronted with the second male directly after the first, what let to a difference between plugged and unplugged females concerning remating. Additionally, with our setup we can not absolutely exclude an effect of seminal substances, which are transferred late during mating additionally to the plugging substance, or the physical attempt to stay with the female to guard the transferred sperm. But since the females in both treatment mated repeatedly, we exclude sperm depletion caused by sperm loss.

Finally, the adaptive value of traits such as plugging that have evolved under natural conditions may be difficult to quantify under laboratory conditions. As stressed by Barker [18] for *C. elegans *plugging might be important under complex natural conditions. Hence, although important in the field, it may be redundant and neutral in the laboratory. But considering that most of the *Caenorhabditis *strains are kept for many generations in the laboratory and still produce plugs despite their presumable cots, there has to be an explanation for their existence.

Our data support some alternative function of a plug. Females without plugs produced fewer offspring, but had the same mating rate as plugged females. This difference is potentially of a beneficial function in which traits that are likely to have evolved under male-male competition alleviate rather than worsen the collateral damage of multiple mating to females. Our design does not allow us to speculate on why and how exactly a plug provides a benefit to a female *C. remanei*.

Compared to other organisms, where beneficial substances evolved directly to convince a female to mate or to use the sperm of a certain male, here we suggest another scenario: The copulatory plug of *C. remanei*, which most likely evolved under sperm competition conditions, turned out to be beneficial for the female. Therefore plugs could still persist, even though their original function could partly be lost. Other studies usually focus on the effects of plugging on paternity, but maybe the effects for the females are more beneficial than expected. In our opinion, copulatory plugs and beneficial substances can not be clearly separated from each other, at least not in *C. remanei*. This topic merits future attention.

## Conclusions

We manipulated plugging behaviour in *C. remanei *and found that plugging does not influence female attractiveness or mating rate, but increases female fitness. We conclude that a function in male-male competition may not be as straightforward as generally expected since we did not find any evidence for a sexually antagonistic function. Instead, a plug may represent a male "gift" to the female, with as yet unknown positive nutritional or physiological effects.

## Methods

### Model organism

*Caenorhabditis remanei *is a bacteria-feeding soil nematode. It is one of several described gonochoric species in the genus *Caenorhabditis *[32,33]. Recent studies suggest that members of the genus *Caenorhabditis *occur on rotting fruit, snails and isopods, the latter possibly functioning as a vector [34]. *C. remanei *is found in nutrient- and microorganism-rich habitats such as compost [35].

*C. remanei *has a short life cycle of about 2.5-3 days at 20°C. The development to sexual maturity includes 4 larval stages (L1-L4). In L4 sexual differentiation is possible under a stereomicroscope. On the second day after hatching, both sexes reach adulthood and start to copulate with multiple partners (own observation).

The female vulva is located in the middle of the female's body. In contrast to females, which have a pointed tail end, males have a ray-stabilized fan at the caudal end. The male genital opening is located at the proximal base of this fan. In this study we used *C. remanei *strain SB 146 provided by the Caenorhabditis Genetics Center, University of Minnesota, MN, USA.

### Experimental setup

Worms were cultivated on 9 cm dishes with NGM (Nematode Growth Medium, 1.5 l water, 51 g Agar, 3.75 g Peptone, 4.5 g NaCl, 1.5 ml Cholesterol/Ethanol (5 g Cholesterol in 1 l 95% Ethanol), 1.5 ml 1 M MgSO_4_, 37.5 ml 1 M KPO_4_, 1.5 ml 1 M CaCl_2_) dishes inoculated with 1000 μl *Escherichia coli *(OP50) solution as food source. For the experiment, we used Petri dishes with a diameter of 3 cm and an amount of 40 μl *E. coli*, which results in a 1 to 1.5 cm food lawn.

Three days before the experiment started worms were age-synchronized. This involves killing all living worms using a 1:1 solution of 5 M NaOH and sodium-hypochloride (12%) and washing off all eggs, which are unaffected by this treatment. Two days later, juvenile L4 females were isolated before they reached maturity in order to obtain virgin focal females. Males stayed in the mixed cultures and were allowed to mate freely to assure a mating eagerness that coincides with that of an average male from the culture. Following this procedure males and females were synchronized for the three experimental days.

Virgin focal females were divided into two treatments: (1) unplugged females, in which mating was interrupted after sperm transfer and (2) plugged females, in which mating was left undisturbed, but followed by a sham treatment (see below). A new mating trial started with the transfer of a focal female to a new 35 mm plate. A (non-virgin) male from the mixed culture was added at a fixed distance of 9 mm to the female. Both individuals were placed on the *E. coli *lawn.

In the unplugged treatment, males were not allowed to produce a plug after insemination. During pilot experiments, we established that CO_2 _can be used to anaesthetize nematodes. As soon as CO_2 _is streaming onto the NGM plate, worms stop moving immediately and can easily be manipulated. In pilot experiments, we confirmed that CO_2_-treated worms did not show negative effects of the treatment relative to untreated worms.

In the unplugged treatment, we anesthetized couples using CO_2 _immediately after completed sperm transfer (Figure 1). Subsequently, we separated male and female using a worm picker, which was constructed by attaching a hair to a Pasteur pipette. We only touched the male and pulled it away from the female softly. This happened after spicule retraction. In some cases the position of the spicules was not visible while the worms were still in mating position, but after we anesthetized and removed the male the spicules we always found retracted in the male's body. In the plugged treatment, we anesthetized the female after copulation for 8 s, which is equal to the mean duration of CO_2 _exposure in the unplugged treatment as determined in pilot experiments. This technique was trained in pilot studies for both treatments so that all experimenters were excellently prepared. Treatments were randomized in time and across observers.

### Measurements

In order to compare female attractiveness between the unplugged and plugged treatment, we recorded the time between the experimental start (both worms placed in the same Petri dish) to the first contact of male and female. Even though the first encounter did not always lead to genital scanning, we assume this measurement to be representative for pheromone production and attractiveness of the female. We also measured the time from first contact to insertion of the spicules, because, in *C. elegans *[29], the time the male needs for scanning the vulva is linked to female receptiveness.

When copulation was terminated or in cases without copulation after 30 min, the female was transferred to a new plate and the male was killed. This procedure was repeated on three consecutive days, six times per day, with a minimum resting time of 1 h between mating trials. Taken together, every female had the possibility of mating 18 times.

Individual fitness was estimated using three parameters: (1) fecundity, defined as the number of eggs produced during the first three reproductive days during the mating trials, (2) fitness, defined as the total number of adult offspring that hatched from these eggs and (3) fertility, defined as hatching rate: the number of offspring divided by the number of eggs, i.e. a measure for reproductive efficiency. To measure these three parameters, we counted eggs on each dish after removal of the female. Three days later the hatched offspring was frozen and counted later.

### Statistics

Since some females died in both treatments during the experimental days (5 for the plugged, 7 for the unplugged treatment) and some worms escaped over night (2 for the plugged, 5 for the unplugged treatment), we solely analyzed individuals that stayed in all 18 sessions of possible matings. The sample size in all treatments and measurements was 24. Individuals with missing data points for specific measurements were excluded from specific comparisons. This was the case for unplugged females for time from contact to mating (n = 22) and for the fecundity measurements (n_unplugged _= 23, n_plugged _= 22) (see Results). The treatments were compared with a Wilcoxon rank sums test for non-parametrical data using JMP 7.0.

## Competing interests

The authors declare that they have no competing interests.

## Authors' contributions

The experiment was designed and discussed by NT, TG, CG, JK, JFP, DS and NKM. Data acquisition, analysis and drafting the manuscript was done by NT, CG, JK, JFP and DS with critical revising and major improvements by TG and NKM. All authors read and approved the final manuscript.
